# Quantitative and Discrete Evolutionary Changes in the Egg-Laying Behavior of Single *Drosophila* Females

**DOI:** 10.3389/fnbeh.2019.00118

**Published:** 2019-05-29

**Authors:** Lasse B. Bräcker, Christian A. Schmid, Verena A. Bolini, Claudia A. Holz, Benjamin Prud’homme, Anton Sirota, Nicolas Gompel

**Affiliations:** ^1^Fakultät für Biologie, Biozentrum, Ludwig-Maximilians Universität München, Munich, Germany; ^2^Aix-Marseille Université, CNRS, IBDM, Institut de Biologie du Développement de Marseille, Campus de Luminy Case 907, Marseille, France; ^3^Bernstein Center for Computational Neuroscience Munich, Faculty of Medicine, Ludwig-Maximilians-Universität München, Planegg-Martinsried, Germany

**Keywords:** *Drosophila*, behavior, ethogram, evolution, oviposition, decision making, egg

## Abstract

How a nervous system assembles and coordinates a suite of elementary behavioral steps into a complex behavior is not well understood. While often presented as a stereotyped sequence of events, even extensively studied behaviors such as fly courtship are rarely a strict repetition of the same steps in a predetermined sequence in time. We are focusing on oviposition, the act of laying an egg, in flies of the genus *Drosophila* to describe the elementary behavioral steps or microbehaviors that a single female fly undertakes prior to and during egg laying. We have analyzed the hierarchy and relationships in time of these microbehaviors in three closely related *Drosophila* species with divergent egg-laying preferences and uncovered cryptic differences in their behavioral patterns. Using high-speed imaging, we quantified in depth the oviposition behavior of single females of *Drosophila suzukii, Drosophila biarmipes* and *Drosophila melanogaster* in a novel behavioral assay. By computing transitions between microbehaviors, we identified a common ethogram structure underlying oviposition of all three species. Quantifying parameters such as relative time spent on a microbehavior and its average duration, however, revealed clear differences between species. In addition, we examined the temporal dynamics and probability of transitions to different microbehaviors relative to a central event of oviposition, ovipositor contact. Although the quantitative analysis highlights behavioral variability across flies, it reveals some interesting trends for each species in the mode of substrate sampling, as well as possible evolutionary differences. Larger datasets derived from automated video annotation will overcome this paucity of data in the future, and use the same framework to reappraise these observed differences. Our study reveals a common architecture to the oviposition ethogram of three *Drosophila* species, indicating its ancestral state. It also indicates that *Drosophila suzukii*’s behavior departs quantitatively and qualitatively from that of the outgroup species, in line with its known divergent ethology. Together, our results illustrate how a global shift in ethology breaks down in the quantitative reorganization of the elementary steps underlying a complex behavior.

## Introduction

The diversity of complex animal behaviors is perceptible in the most subtle differences of innate behaviors between closely related species. Wolves and dog breeds looking like wolves are unmistakingly identified by a suite of different elementary behaviors (Heberlein et al., 2016). Bird species of the same genus can be identified on the sole basis of their songs (Slabbekoorn and Smith, 2002). The same holds true for cicadas (Young, 1972). *Drosophila* flies from different species engage in distinct courtship rituals (Spieth, 1952). Strikingly different innate complex behaviors, such as feeding or reproductive behaviors, stem from simple differences in the suite of behavioral steps that together constitute the overall action (Spieth, 1952).

These differences have been described and proposed to be adaptive to particular niches or to evolve under specific selection regimes in a number of cases (Houde and Endler, 1990; Kelley and Endler, 2012; York and Fernald, 2017). To understand the genetic and neuronal change underlying their evolution, it is first necessary to define an experimental framework where the behavioral changes between species can be measured and compared. In the case of complex behaviors produced from the assembly of many elementary steps, the variation between species may be qualitative and quantitative. The quantitative variation itself may encompass, not only the frequency or duration of certain steps, but also their temporal distribution in the entire complex behavior. Therefore, the identification and description of changes in a complex behavior, a prelude to understanding neuronal circuit evolution, call for a quantitative behavioral framework.

To investigate the origin of differences in an innate behavior, in a system where it will later be possible to unravel the genetic and cellular changes, we are focusing on a defined reproductive behavior, egg laying, in *Drosophila* (Yang et al., 2008; Laturney and Billeter, 2014; Karageorgi et al., 2017). Choosing a suitable substrate for ovipositioning is a critical event in the lifetime of a holometabolic insect. It greatly influences the chances of survival of its offspring, especially in species where larval stages are less mobile. The ability of a female to predict the quality of an oviposition substrate will determine the survival of her offspring. Several recent examples have shown that innate female preferences can evolve with transitions to new ecological niches (Matsuo et al., 2007; Billeter and Wolfner, 2018).

Flies of the genus *Drosophila* also show a broad diversity of egg-laying behaviors, with many species laying exclusively on one or a few given species of plants, while others are generalists (Kambysellis and Heed, 1971; Markow and O’Grady, 2005; Ort et al., 2012). The preferences often have to do not with the plant *per se*, but its stage of maturation, such as the ripening or decay state of a fruit (Kambysellis and Heed, 1971; Markow and O’Grady, 2005; Karageorgi et al., 2017). The comparison of divergent behaviors in this group of flies has been a focal point in the field, because these species can often be raised in the lab, and because the comparative work can be anchored with the model species *D. melanogaster*, benefiting from considerable efforts to link genes to neuronal circuits and to behavior. However, the majority of these studies has focused on differences in the animals sensory perception influencing the final outcome of a choice behavior rather than on the details of egg-laying behavior itself (Joseph et al., 2009; Yang et al., 2009; Joseph and Heberlein, 2012; Azanchi et al., 2013; Gou et al., 2014).

We have recently shown that *D. suzukii*, an invasive pest species causing damages to cultivated fruits, and a close relative to *D. melanogaster*, had evolved novel preferences to select an oviposition site (Karageorgi et al., 2017). *D. suzukii* females prefer to lay their eggs in ripe or ripening fruits, while other species, including *D. melanogaster* and *D. biarmipes* prefer decaying fruits to lay their eggs. These preferences result from changes in multiple sensory modalities, including olfaction, taste and touch, and are measured by the number of eggs laid at one site relative to another. How a female concretely probes a potential oviposition site is only superficially described (Yang et al., 2008). To understand this evolutionary variation, and ultimately identify specific neuronal changes underpinning it, we first need to identify potential qualitative and quantitative changes in the individual behaviors, including changes in the presence, frequency, duration and relative arrangement in time of the elementary steps composing the complex behavior. Only then can we try to link genes or neurons to behavioral changes. Previous work has described the selection of an oviposition site in *D. melanogaster* as a looping sequence of behavioral components, including probing the substrate with the ovipositor, resting and searching (Yang et al., 2008). This seminal work, though, did not break down the components further into their elementary steps involving specific appendages, nor did it explore the temporal relationships of the components. Our own preliminary observations suggested that there was no strict looping sequence of elementary behaviors. We, therefore, set out to examine the egg-laying behavior of *D. suzukii* quantitatively, at the level of single flies and at a higher spatial and temporal resolution. We also decided to compare it, in an evolutionary nutshell, to that of *D. melanogaster* and* D. biarmipes*. Interestingly, our quantitative comparisons of single fly behaviors first show, in the form of ethograms and statistical analysis, that the structure of the egg-laying behavior is conserved across species. Importantly, beyond this shared architecture, our work reveals an evolutionary change in the mode of substrate evaluation in *D. suzukii*, compared to the other species. This mode of substrate evaluation is characterized by quantitative changes in the temporal relationships between elementary behavioral steps, referred to as microbehaviors. It may reflect at the level of single flies the behavioral particularities identified in fly groups (Karageorgi et al., 2017).

## Materials and Methods

### Fly Rearing

Flies were reared at 22°C and 50% rH with a 12/12 h light and dark cycle. We used the following stocks, as in Karageorgi et al. (2017): *D. melanogaster*: *Canton S*; *D. biarmipes*: an isofemale line collected from Bangalore, India; *D. suzukii*: an isofemale line from France (Alpes-Maritimes). Flies were reared on a standard corn meal medium and separated at the age of 5–6 days after emergence for experiments.

### Single Fly Egg-Laying Assay

**Figure 1:**
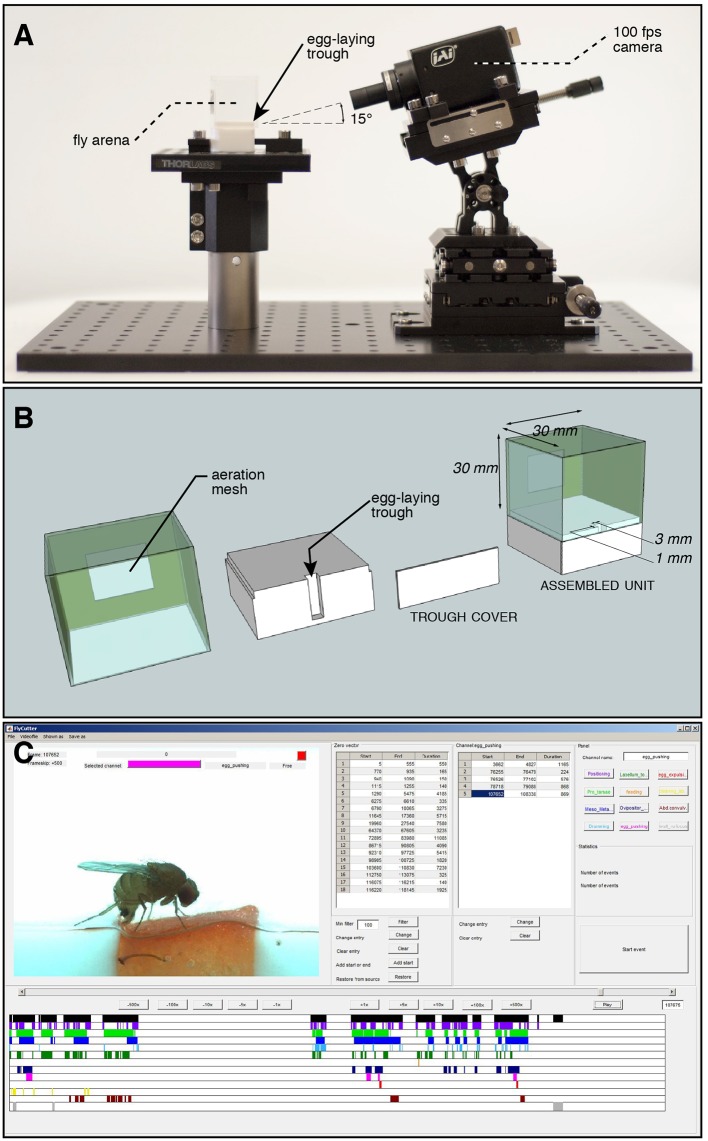
Set-up for recording egg-laying behavior of individual flies. **(A)** Stage overview: a cubic chamber containing a fertile female *Drosophila* is positioned on a pedestal. It contains a trough, the single possible site for laying eggs, facing a high-speed camera positioned at a 15° angle to increase depth of field perception. **(B)** Exploded view drawing of the arena to record individual fly oviposition. A piece of fruit is fitted into the trough before each assay. The surface of the arena is hard plastic, unsuitable for oviposition. **(C)** Screen capture of our custom annotation software used to mark start and end frames of 11 microbehaviors identified around the laying of an egg. The output is a spreadsheet for each annotated video, reporting the times of each annotated event.

Flies were set in a cubic plastic chamber (Figures 1A,B) with an arena containing a single oviposition site onto which a video camera was focused. Two hours before the experiment, groups of flies were transferred to small plastic vials containing only tissue paper and water. This treatment ensured that females did not lay eggs just before the experiment. Single females from these groups were then placed into the arena that contained a 3 × 1 mm piece of a commercially available strawberry as egg-laying substrate. Only the fruit surface (skin), not the fruit flesh was exposed to the fly. *D. suzukii* and *D. biarmipes* were recorded for 20 min after introduction into the arena. *D. melanogaster* females showed a delayed interaction with the medium. To compensate, recording was extended to 1 h after introduction, which led to the successful capture of egg-laying events. The last 20 min were annotated for this species. This corresponds to at least one egg-laying event per video.

### Recording of Fly Behavior

Videos were recorded with a high speed color camera (JAI RMC-6740 GE, IMACO) and a 0.5× objective (Opto Engineering), mounted at a 15° angle on a micromanipulator platform (Thor Labs). To achieve homogenous lighting, a column of 5 cm thick white plexiglas was manufactured to surround the arena, and at the top an array of white LEDs were used for illumination.

### Annotation of Fly Behavior

Eight videos of each species were first processed with a custom software in MATLAB to identify all the frames that showed the fly in focus of the camera. This was achieved by thresholding dark pixels belonging to the shape of the fly body based on intensity, and calculating all frames with a high enough number of these pixels. The resulting lists of frames containing fly activity were manually checked and corrected if necessary, ultimately forming a list of all frames of a video showing fly activity. Within these video segments, which we defined as “active time,” 11 fly behaviors (Table 1) were manually annotated by recording start and end frames of each behavior event and storing these values in respective lists. Alternatively, for the temporal analysis these data can be treated as time series of discrete events (beginning or end) of microbehavior associated with their respective labels (Figure 3A).

**Table 1:** Microbehaviors during *Drosophila* oviposition.

Microbehavior	Description
Positioning	A change in position of the fly body in the horizontal plane (rotation, translation)
Protarsal contact	Protarsae are directly touching the egg-laying substrate
Mesotarsal and/or metatarsal contact	Mesotarsae and/or mesotarsae are directly touching the egg-laying substrate
Abdominal drumming	Upward and downward abdomen movements
Labellum contact	Labellum is directly touching the egg-laying substrate
Feeding	Combination of direct contact with substrate and peristaltic motion of the proboscis indicating food/liquid uptake
Ovipositor contact	Sequence of direct ovipositor contacts with the egg-laying substrate, accompanied by the downward bending of the abdomen
Egg pushing	Pushing an egg through the oviduct, causing it to contract and expand in bouts
Egg expulsion	An egg detaching from the ovipositor
Grooming	Cleaning the head with the first leg pair or cleaning the abdomen with the third leg pair
Abdominal convulsion	Compression and relaxing of the abdomen without abdomen motion relative to the rest of the body

**Figure 2:**
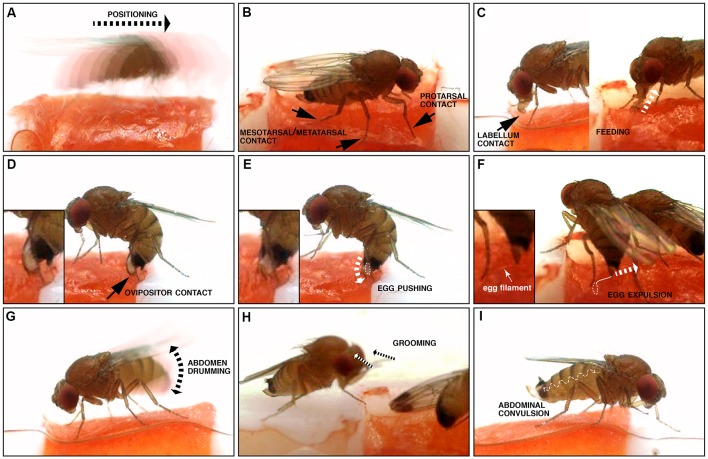
Illustration of microbehaviors occurring before and during oviposition. **(A)** Positioning, where the fly relocates (motion is indicated by the dotted arrow). **(B)** Tarsal contacts; note that all tarsae are not necessarily simultaneously in contact with the substrate. **(C)** Labellum contact and feeding: both involve contact of the substrate with the distal mouthparts, the labellum. Labellum contact is limited to this, while feeding is accompanied by peristaltic movements of the proboscis corresponding to food uptake (depicted by the dotted arrow), and an open labellum. **(D)** Ovipositor contact: this microbehavior happens along with a characteristic bow of the abdomen. **(E)** Egg pushing: similar to ovipositor contact, but the ovipositor is inserted into the substrate, an egg is engaged into the egg canal (dotted white line), and convulsive movements of the ovipositor are visible (depicted by the dotted arrow). **(F)** Egg expulsion: the egg is separated from the female’s body, usually inserted into the substrate (dotted white line), but leaving the egg filaments visible (solid white line). **(G)** Abdomen drumming, where the abdomen as a whole is moved up and down (the double arrowhead indicates the direction of the abdominal movements). **(H)** Grooming: the fly cleans its head or its body with its legs, in a stereotypical brushing movement (dotted arrows). **(I)** Abdominal convulsion: the wiggly dotted line depicts the direction of the abdominal movements, when the female’s abdomen contracts and extends like an accordion.

**Figure 3:**
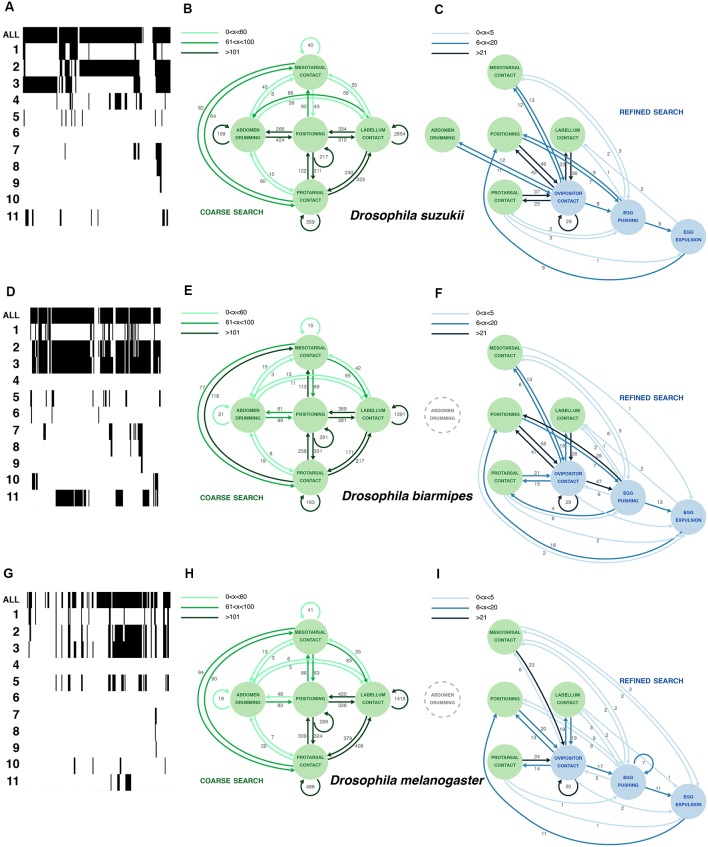
Comparative oviposition ethograms for *D. suzukii* **(A–C)**, *D. biarmipes* **(D–F)** and *D. melanogaster* **(G–I)**. **(A,D,G)** Selected examples of activity traces (20 min) for a single *D. suzukii* **(A)**, *D. biarmipes* **(D)** or *D. melanogaster* **(G)** female showing total activity when a fly engages the substrate as well as counts of 11 microbehaviors (1: *positioning*; 2: *protarsal contact*; 3: *meso-, metatarsal contact*; 4: *abdomen drumming*; 5: *labellum contact*; 6: *feeding*; 7: *ovipositor contact*; 8: *egg pushing*; 9: *egg expulsion*; 10: *grooming*; 11: *abdominal convulsion*). Arrows (in **B,C,E,F,H,I**) were plotted based on total occurrence of start-to-start transitions between microbehaviors. **(B,E,H)** Plotting the most frequent transitions (dark green arrows) reveals a common search loop connecting several microbehaviors that we interpret as sensory sampling of the substrate. Numbers next to the arrows denote the total number of transitions observed in across eight annotated videos per species (3 × 8 videos together). Arrows were plotted based on total occurrence of start-to-start transitions between microbehaviors. **(C,F,I)** Plotting transitions between the three microbehaviors that involve the ovipositor reveals a sequence of behaviors that ends with egg laying and is embedded in the continuous sampling of the substrate. The connection between these ovipositor-related microbehaviors and sampling microbehaviors is much weaker in *D. melanogaster*.

### Analysis and Statistics

#### Ethogram Construction

Start-to-start transitions between microbehavior events were defined as follows: the occurrence of a start frame of an event after the start frame of a different event within a 10 s window. This included transitions between events of the same microbehavior as well as the possibility of multiple transitions occurring after the same event.

#### Microbehavior Duration Analysis

Measurements were based on frames as a unit for time passed. With a constant recording speed of 100 frames per second, one frame equals to one centisecond of elapsed time. The relative time spent on a microbehavior type for each species was calculated by normalizing the total duration of frames in which a fly commits to this microbehavior to the overall active time of this individual. Active time represents all frames in which the fly is fully in focus of the camera (Figure 1C). As a consequence of the specifications of the arena and the chosen field of view, this results in active time representing the time a fly interacts with the substrate. Groups of eight individuals per species were then compared using a one-way ANOVA and a Bonferroni corrected *post hoc* test.

#### Temporal Structure of Transitions Between Microbehaviors

Temporal relationship between transitions to each microbehavior was analyzed using time-lagged cross-correlation analysis of discrete event time series based on microbehavior onset times. This analysis provides normalized probability of microbehavior state transitions at different time lags between state onsets. Data were treated in two ways: one, which, as in ethogram analysis, combined individual fly time series, and the other, which took each fly time series separately. Time bin for cross-correlogram estimation was set to 1 s. All microbehavior onset time series were “whitened” by removing bouts of events of the same type with inter-event interval of less than 2 s, thus making time series more consistent with Poisson assumption required for proper interpretation of the temporal cross-correlation functions. The assumption of a Poisson distribution of independent microbehavior events for longer time scales is also, however strong, and requires caution. In fact, when examining activity traces (Figures 3A,D,G), it is obvious that microbehaviors of one type often happen in trains, and that distinct microbehaviors are often temporally clustered (consecutive, overlapping or nested). Cross-correlation values were normalized to asymptotically converge to 1 under independence model. For single fly analysis, we excluded flies that had fewer than five ovipositor contact events to provide minimal statistical power to the estimator. Group-average cross-correlation functions were computed as a bootstrap across group sample (flies) giving rise to unbiased mean and confidence intervals. Temporal asymmetry of the group-average cross-correlation functions was quantified as a normalized change in cross-correlation values between 20 s following and preceding zero lag. Significance was tested using two-sided sign test.

## Results

### Eleven Microbehaviors Describe Substrate Exploration and Oviposition of a Single *Drosophila*

To observe the egg-laying behavior of individual flies, we built chambers where the only suitable spot for oviposition was a 3 mm^2^ area filled with a ripe piece of strawberry, an egg-laying substrate well accepted by all three species in no-choice assays (Karageorgi et al., 2017), the rest of the arena being hard plastic (Polytetrafluoroethylene). After introducing a single, fertilized female into the chamber, we recorded all behaviors displayed by the fly on the fruit and in its immediate vicinity with a high temporal resolution of 100 frames per second (Figures 1A,B). For each individual fly, we analyzed 20 min of video, starting with the introduction of the fly to the arena, a sufficient time to capture at least one event of egg laying. We chose this 20-min window to ensure a saturated coverage of all relevant behaviors in our data. This is reflected by sections within our videos, in which the fly does not interact with the medium. In the case of *Drosophila melanogaster*, though, 20 min did not suffice and we instead analyzed the last 20 min of a 1 h video after introduction. We used a custom, frame-by-frame video annotation program (Figure 1C) to manually extract quantitative behavioral information from this data set (Supplementary Table S1). Overall, we annotated oviposition behavior for eight individual females of each of the three species *D. melanogaster*, *D. biarmipes* and *D. suzukii*. Our annotation was based on a catalog of elementary behavioral patterns we termed microbehaviors. These represent the simplest recurring patterns that all of the recorded data could be divided into. To avoid any bias in the definition of this catalog, we decided to include all microbehaviors that we could consistently recognize (Table 1, Figure 2). They can be grouped into three categories.

The first category comprises microbehaviors where sense organs directly touch the egg-laying substrate, with the exceptions of contact involving the ovipositor, which we arbitrarily placed in the next category (see hereafter). Microbehaviors in this first category allow the fly, in principle, to collect information relating to the texture and chemical composition of the substrate. This category includes *positioning* (Figure 2A), *protarsal contact* (Figure 2B), *meso/metatarsal contact* (Figure 2B), *labellum contact* (Figure 2C) and *feeding* (Figure 2C). We included this latter microbehavior in this first category because it leads the fly to be exposed to a new set of sensory information. The second category consists of microbehaviors involving the ovipositor. It includes *ovipositor contact* (Figure 2D), *egg pushing* (Figure 2E) and *egg expulsion* (Figure 2F). Finally, we identified three additional stereotyped motor patterns: *abdomen drumming* (Figure 2G), *grooming* (Figure 2H) and *abdominal convulsion* (Figure 2I), which did not fall into any of the previously described categories. The rest of our analysis does not explore *abdominal convulsion* and *grooming* further. While *grooming* was described as part of a looping sequence of egg laying in a previous study (Yang et al., 2008), this microbehavior was rare and inconsistently displayed by individual flies of the same species in our assay (Supplementary Table S1). *Abdominal convulsion* on the other hand was both difficult to make sense of and displayed at similarly low rates by all three species (Supplementary Figure S1). Together, all these microbehaviors can be used to describe all observable fly actions during the annotated 20 min when a fly explores an oviposition site, and lays an egg.

### An Ethogram of Oviposition for *Drosophila suzukii*

Our annotated videos have 11 distinct tracks (one per microbehavior), displaying the start and end frames of each microbehavior occurrence, which we refer to as an “event” (Figure 1C; Supplementary Table S1). To construct an ethogram representing the collected data (Figure 3; Supplementary Table S1), we calculated the number of transitions between each possible combination of microbehaviors, including repeated occurrence of the same microbehavior. We decided to count as a transition the occurrence of the start frame of the next microbehavior following a given event in the same or any other microbehavior. This method allowed us to detect sequences of events that were either separated in time, or overlapping or nested. The choice of this start-to-start method, as opposed for instance to an end-to-start method, is especially important for including transitions to microbehaviors of the contact category. Typically, a fly would start to contact the egg-laying substrate, for instance with the protarsae, and meanwhile initiate other microbehaviors, without ending the *protarsal contact* yet.

We first used the start-to-start transitions of consecutive microbehavior events derived from eight individual *D. suzukii* videos to build an ethogram, focusing on eight microbehaviors (leaving *abdominal convulsion*, *grooming* and *feeding* out, because these microbehaviors were rare, inconsistent between flies, or difficult to interpret). A graph depicting all transitions as arrows connecting microbehaviors as nodes is obscured by the density of the network (not shown). To make sense of our analysis graphically, we resorted to another strategy. We noted that a few transitions occurred at a much higher frequency (hundreds to thousands of occurrences in the eight concatenated *D. suzukii* videos; Figure 3B, Supplementary Table S1) than the rest of the transitions (units to tens), and we started by plotting these. They define a loop involving five microbehaviors densely connected to *positioning* (Figure 3B). We consolidated this plot by adding all transitions of lower frequencies that connect these five microbehaviors. Several microbehaviors in this loop repeat themselves consecutively at a high frequency. This is strikingly the case with *labellum contact* occurring in bouts with up to 2,654 repetitions. In these bouts, the fly extends her proboscis, samples the substrate surface with her labellum repeatedly, and often terminates the bout by repositioning. We interpret this loop as a coarse search, whereby a fly explores the surrounding space intensely, with lots of repositioning, before selecting a potentially suitable area for her egg.

We then separately plotted all remaining transitions (Figure 3C) and realized that they connect all ovipositor-relating microbehaviors to the previous five microbehaviors, but with transition frequencies that are overall much lower (1–49 occurrences in the eight concatenated *D. suzukii* videos; Figure 3C, Supplementary Table S1). This new plot defines a second loop that we interpret as a refined search, densely connected to *ovipositor contact*, whereby the local substrate exploration is limited (much fewer repositioning), and the ovipositor, covered with sensory organs, is now directly contacting the substrate. This can bring the fly to a successful oviposition.

Together these two loops form an ethogram describing a non-linear series of microbehaviors that can converge to the deposition of an egg. These results contrast strikingly with the sequential loop that was described for *D. melanogaster* oviposition (Yang et al., 2008). This leads us to ask how *D. melanogaster* females, in our assay, differ from *D. suzukii* females during the selection of a site for egg laying.

### A Robust Architecture for the Oviposition Ethogram

*D. suzukii* females prefer to lay their eggs on ripe fruits, unlike females of closely related species such as *D. melanogaster* or *D. biarmipes* (Karageorgi et al., 2017). We wondered if this difference in substrate preference was accompanied by a change in their egg-laying behavior itself. To compare how single flies actually proceed to finding an oviposition site, and eventually lay an egg, we leaned on the egg-laying ethogram that we have built for *D. suzukii*, and leaning on video annotations of these species (Supplementary Table S1) likewise built ethograms for *D. melanogaster* and *D. biarmipes*. We first observed a striking qualitative similarity between the behavior of all three species. The catalog of microbehaviors found in *D. melanogaster* and *D. biarmipes* was identical to that of *D. suzukii*. Each microbehavior can unambiguously be recognized, regardless of the species, and no additional microbehaviors were to be seen in *D. melanogaster* and *D. biarmipes* females. We, therefore, applied the methodology described above to build egg-laying ethograms for these two additional species. The results, the architecture of the ethograms and the overall pattern of connectivity are very similar between these species (Figure 3), suggesting a robust behavior.

### Evolutionary Change in Egg-Laying Behavior

In spite of a conserved architecture, these ethograms offer notable differences. First, there are differences in the pattern of connectivity between microbehaviors, as illustrated with *abdomen drumming*. It is strongly connected to the primary loop of *D. suzukii* but disconnected from this loop in *D. biarmipes* and *D. melanogaster*, which display much less transitions to and from *abdomen drumming*. This surge of transitions from and to *abdomen drumming* in *D. suzukii* is probably due to the more frequent occurrence of this microbehavior in this species. In the secondary search loop, as for the primary search loop, most transition frequencies are similar between all species. Only *D. suzukii* displayed more transitions between *abdomen drumming* and *ovipositor contact*.

**Figure 4:**
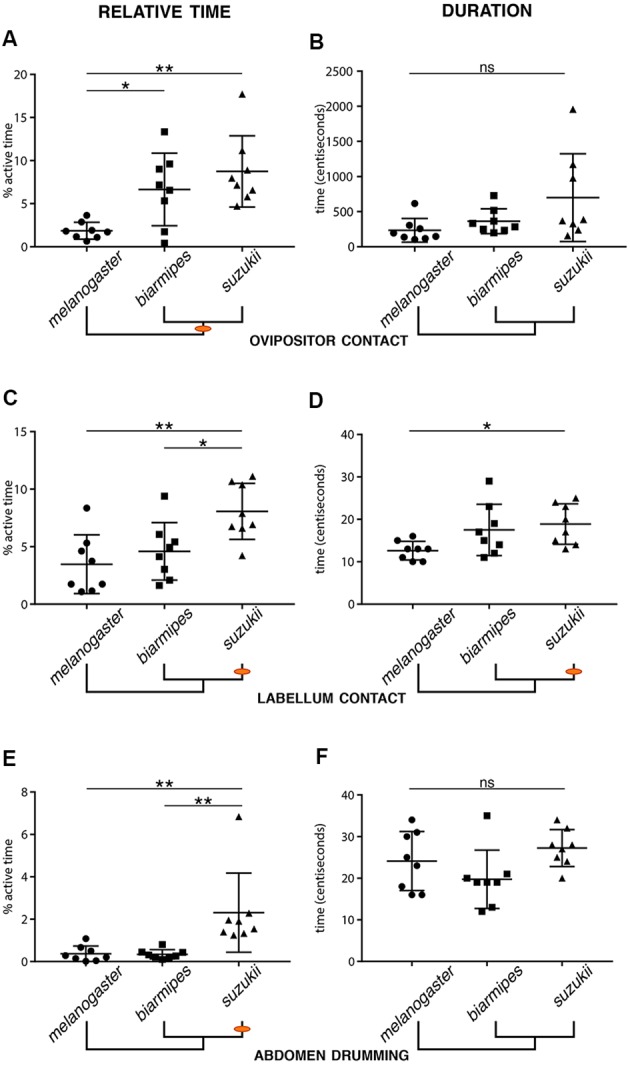
Evolutionary changes in the relative time and duration of specific microbehaviors. Relative oviposition time (left column) or average duration (right column) for the following microbehaviors: *ovipositor contact* **(A,B)**, *labellum contact* **(C,D)**, and *abdomen drumming* **(E,F)**. Orange dots on the phylogenetic trees indicate the origin of a divergence (Error bars represent SEM, *n* = 8, stars indicate significant differences between two groups; ns: non-significant). **(A,B)** Both *D. suzukii* and *D. biarmipes* spend significantly more time on *ovipositor contact* when interacting with the substrate than *D. melanogaster* **(A)**. While not significant, *D. suzukii* displayed a tendency for longer ovipositor contacts **(B)**. **(C,D)** *D. suzukii* spends more active time on *labellum contact* than *D. biarmipes* and *D. melanogaster* **(C)**. The duration of the bouts of labellum contact is also significantly longer in *D. suzukii* than in *D. melanogaster*. **(E,F)** *D. suzukii* spends significantly more time on *abdomen drumming* than both *D. biarmipes* and *D. melanogaster*, but displays no increase in the average duration of drumming events.

We also identified specific quantitative differences when comparing the total number of observed microbehavior events, their average duration, and the percentage of active time spent on each microbehavior (defined as total time displaying a specific behavior divided by time spent in contact with the substrate; Figure 4). First, we compared behavioral parameters related to the primary loop. We found changes in several microbehaviors related to sensory sampling of the substrate for *D. suzukii* compared to *D. melanogaster*. We found that *D. suzukii* females spent more of their active time contacting the substrate with their labellum than the two other species. While the absolute number of *labellum contact* events was nearly unchanged, we found an increase in relative time and average event duration when comparing *D. suzukii* to *D. melanogaster* (Figures 4C,D). Likewise, *D. suzukii* females spent a larger proportion of their active time repositioning themselves and also showed an increase in the duration of protarsal contacts (Supplementary Figure S2). We interpret these results as an enhanced primary search loop exhibited by *D. suzukii*, potentially enabling this species to collect more sensory information about the substrate and with a finer spatial resolution. Interestingly, when focusing on *D. biarmipes* across all statistical comparisons, we find that this species is not consistently similar to either of the other two species. Instead, we find microbehaviors for which *D. biarmipes* is behaving similar to *D. suzukii*, such as the relative time spend on *ovipositor contact*, and others for which *D. biarmipes* is behaving similar to *D. melanogaster* (Figure 4; Supplementary Figure S2). This lack of clear grouping across all parameters places *D. biarmipes* behavior in an intermediate position, in line with what we had found for the oviposition site preferences of this species (Karageorgi et al., 2017).

In the secondary loop, the refined search, we found several similarities between *D. suzukii* and *D. biarmipes* that distinguish them from the outgroup species, *D. melanogaster*. Both species spent a larger fraction of their active time contacting the substrate with their ovipositor (Figure 4A), or pushing the egg (Supplementary Figure S2), potentially collecting more information on the local substrate just prior to, and during oviposition. While these similarities may simply reflect the phylogenetic proximity of *D. suzukii* and *D. biarmipes*, they may also sign a profound difference in the selection of an oviposition site, a higher stringency in the choice than *D. melanogaster*. In this secondary loop, some behavioral parameters were unique to *D. suzukii*, such as a longer duration of *ovipositor contacts* or *egg pushing* (Supplementary Figure S2), or *egg expulsion* (Supplementary Figure S2), compared to *D. biarmipes*. These increased times may point to a higher selectivity of *D. suzukii* and may relate to the fact that *D. suzukii* females, unlike females of the other species, lay their eggs into the substrate.

As a side note, perhaps more difficult to interpret, we found that *D. suzukii* engaged in significantly more *abdomen drumming* events compared to *D. biarmipes* or *D. melanogaster* (Figure 4E), yet the duration of these bouts of *abdomen drumming* remained the same for all species (Figure 4F). The increased amount of *abdomen drumming* events also explains why this node is well connected to several other behavioral nodes in *D. suzukii*’s ethogram (Figures 3B,C). The functional significance of *abdomen drumming* in the context of oviposition remains an open question.

### Temporal Cross-Correlation Analysis of Microbehavior Transitions Suggests Potential Differences in the Exploration Patterns Among *Drosophila* Species

**Figure 5:**
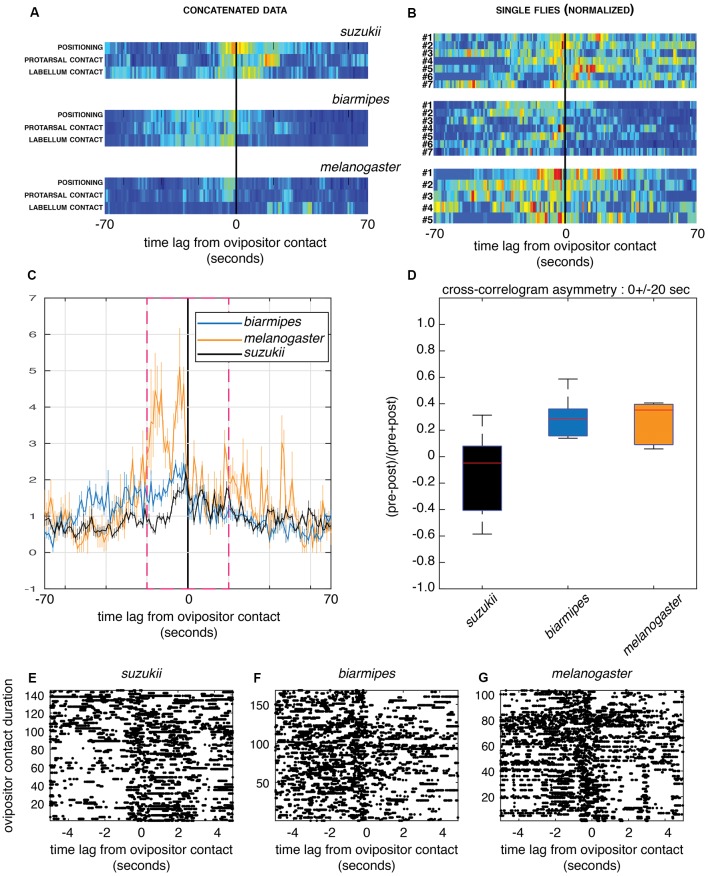
Cross-correlograms between ovipositor contact and other sensory sampling microbehaviors. **(A,B)** Cross-correlograms centered on *ovipositor contact* (0) in a time interval of 140 s for *D. melanogaster*, *D. biarmipes* and *D. suzukii*. The representations, normalized for visualization purpose, are pseudo-color maps of event counts. **(A)** Events detected from all flies for each species group were concatenated and each row represents the cross-correlogram of different sensory sampling microbehaviors to *ovipositor contact*. Note the different asymmetries in the relationship of these microbehaviors for *D. suzukii* compared to the other species. **(B)** Each row represents a single fly for which microbehaviors assumed to be involved in sensory sampling (*positioning*, *protarsal contact*, *labellum contact*) were pooled. The color maps highlight the strong variation in the distribution of sensory sampling microbehaviors, with perhaps a similar trend in the asymmetrical distribution shown in **(A)**. Only animals with a count of at least five events of *ovipositor contact* were used to generate the plots in **(B)**. **(C,D)** Group-averaged cross-correlograms centered on *ovipositor contact* as in **(B)** showing the temporal asymmetry in sensory sampling between the species (**C**; cross-correlation values are normalized to asymptotically converge to one under independence model) and its quantification **(D)** for a 40 s window within which the temporal correlation happens (magenta dashed frame; two-sided sign test, *p* = 1 for *D. suzukii, p* = 0.015 for *D. biarmipes* and *D. melanogaster*; **E–G**) Example of *labellum contact* events, shown as rasters (each dot is a behavior onset) centered on *ovipositor contact* for all flies within each group, **(E)** *D. suzukii*, **(F)** *D. biarmipes* and **(G)** *D. melanogaster*. *Ovipositor contact* events are ordered by their duration in the increasing order along the y-axis. Note the striking difference of asymmetry between *D. suzukii* and the other species for short (first ~100 events across all videos of a single species) contacts.

While the previous analysis provides a comprehensive framework to describe what a fly does in the context of oviposition, it eludes an important dimension of this activity, the relationships between different microbehaviors in time. Cross-correlogram between two discrete event trains of microbehavior onsets provides a quantitative measure of temporal relationship that is not visible in the ethograms. Indeed, while ethograms examine transitions between consecutive events, cross-correlograms examine the relationship between events that may be separated in time by intervening events. Here, we have chosen to focus on a nodal microbehavior, *ovipositor contact*, because it marks the onset of the egg-laying motor program. We have analyzed the temporal dynamics of other events, in particular, those that we can interpret as sensory sampling of the substrate (*positioning*, *protarsal contact* and *labellum contact*), with respect to *ovipositor contact* (Figure 5). Our analysis indicates interesting trends that single out *D. suzukii*, in spite of a noisy dataset due to individual fly variation.

First, we estimated cross-correlograms using the time series of microbehavior onset events concatenated within each species group. The resulting cross-correlogram in a window of ±70 s centered on *ovipositor contact* for single flies (Figure 5A) shows a clear-cut difference between *D. suzukii* and its relatives in the temporal dynamics of sensory sampling relative to the ovipositor contacts. In *D. suzukii* indeed, *ovipositor contact*, on average, precedes substrate probing with other body parts, while in *D. biarmipes* and *D. melanogaster* it tends to end a phase of substrate sampling. This finding indicates that in *D. suzukii*, *ovipositor contact* is a part of sensory sampling bouts, rather than terminating them.

Second, we investigated the variability of the observed temporal dynamics between flies within each species group and computed cross-correlograms of sensory sampling microbehaviors relative to *ovipositor contact* for each fly. Figure 5B shows individual cross-correlograms in a window of 140 s centered on *ovipositor contact* for single flies with three sensory sampling microbehaviors (*positioning*, *protarsal contact* and *labellum contact*) that were pooled to simplify the display. Clearly, small sample sizes of events detected within each fly provide much noisier cross-correlograms. In addition, this analysis highlights high variation between flies of the same species. Nevertheless, group average cross-correlograms (Figure 5C) did show a distinct temporal asymmetry consistent with that observed in Figure 5A. When cross-correlogram asymmetry index is showed per species (Figure 5D), *D. suzukii* is again singled out from its relatives that show, as a group, increased probability of sensory sampling prior to ovipositor contacts. Given small sample sizes within each group and low number of events in each fly, as well as inter-fly variability, quantitative comparison between the groups cannot be properly performed using the data at hand.

Finally, such asymmetry is more apparent when focusing on some microbehaviors and sorting *ovipositor contact* events by their duration. Strikingly, events of *labellum contact* tend to happen more after an *ovipositor contact* in *D. suzukii*, while they tend to precede an *ovipositor contact* in *D. biarmipes* and *D. melanogaster*. This is particularly striking for short contacts when events of ovipositor contact are plotted by their duration (Figures 5E–G). This also suggests two modes of *ovipositor contact*, short and long, the former involved in substrate sampling and the latter linked to *egg pushing* and to the egg-laying motor program.

Together, the cross-correlograms suggest an interesting hypothesis, where the role of *ovipositor contact* would have changed in *D. suzukii* compared to its close relatives *D. biarmipes* and *D. melanogaster*.

## Discussion

We have established a method to reliably record and annotate egg laying in *Drosophila* at a single fly level and with a very precise spatial and temporal resolution. We used this framework to compare how females of the closely related species *D. biarmipes*, *D. melanogaster* and *D. suzukii* may differ in their approach to laying an egg. In particular, as *D. suzukii* is known to prefer ripe fruits over decaying ones for egg laying (Karageorgi et al., 2017), we scrutinized the microbehaviors of its females, wondering if they departed from those of its close relatives.

From the resulting dataset, we derived ethograms depicting the observed transitions between different steps preceding oviposition. This analysis broadens the published description of single fly egg-laying substrate selection (Yang et al., 2008), but also indicates that the sequence described by these authors is, in fact, a network of interconnected elementary behavioral steps. The frequencies of transitions fell in two large bins outlining what we interpret as two search loops, a coarse search where a fly identifies a suitable site and a refined search consisting of a final quality control for the exact position where the egg may end. Strikingly, this behavioral architecture holds across all three species, suggesting an ancestral backbone, in the same way that *Drosophila* male courtship appears to be composed of a suite of simple interconnected events that mostly vary qualitatively (Spieth, 1952).

This conserved architecture of relationships between microbehaviors nevertheless shows significant quantitative variations. First in the frequencies of transitions between specific microbehaviors, but we also noted quantitative differences in the relative time spent on different microbehaviors or in the duration of their occurrences. While the emphasis on microbehaviors such as *abdominal drumming* in *D. suzukii* is difficult to interpret, other differences may sign the evolutionary transition that emerged within this group of species. Indeed, in a recent study where we compared the egg-laying preferences of different *Drosophila* species for different substrates (Karageorgi et al., 2017), we found that *D. melanogaster* shared the preference of a large number of other species for rotten fruits and soft substrates, while *D. suzukii* radically chose to lay on ripe or even unripe fruits, and tolerated much harder substrates. In multiple assays, *D. biarmipes* displayed an intermediate behavior. In the present study, we are not asking the flies to choose between substrates, and in this respect, we cannot directly compare the current results to those of our past work. Nevertheless, here as well, we found that *D. suzukii* often departs from *D. melanogaster* and *D. biarmipes* in aspects of its exploration of the egg-laying substrate (Figure 4).

This trend is also seen, perhaps generalized, in cross-correlation analysis, where in *D. suzukii*, an ovipositor contact appears to elicit further sensory exploration, while it seems to terminate an exploration phase in the other species, representing an attempt of egg laying. This observation emphasizes that the ovipositor, more than a simple structural guide to lay or insert an egg, is also involved in the sensory evaluation of the substrate, similar to the legs and mouthparts. It is, as a matter of fact, covered with external sensory organs, whose evolutionary variation may indicate a concerted evolution with behavior (Atallah et al., 2014).

The cross-correlogram analysis reveals behavioral patterns that the classical representations of behavior would miss. Nevertheless, in the present study, the limited amount of data (small numbers of *ovipositor contact* on which we centered the correlograms) and the small number of replicates (eight annotated videos per species) makes our conclusion vulnerable to biases. The concern is substantiated by the observation of individual variation (Figure 5B). The manual annotation of 24 videos represented an important investment of time. It falls short, however, of providing the amount of data we would need to strengthen our conclusion and study quantitatively further aspects of the ethogram, such as characteristic temporal parameters or dependencies of the ethogram structure on the hidden variables reflected in the duration of microbehaviors, their specific sequences etc. The only realistic alternative that we envision for the future of our project is to implement a system of automated annotation, such as what was recently described in Mathis et al. (2018).

The assay and analysis that we describe here pave the way for dissecting the relationship between microbehaviors and the neurons that control them, but also the neurons that coordinate the transitions between microbehaviors. Indeed, we can use this system to compare not only different species but also mutants or transgenic animals where subset of neurons are genetically controlled. Likewise, a simple adaptation of our behavior arena will make it possible to offer the flies a choice between two substrates, and therefore to ask how group preferences measured by the number of eggs laid on one or the other substrate, break down at the level of single flies and their microbehaviors.

## Data Availability

All datasets generated for this study are included in the manuscript and/or the Supplementary Files.

## Ethics Statement

The research was conducted with *Drosophila* flies and did not require permit or approval from an ethics committee. It did not involve endangered species either.

## Author Contributions

LB, BP and NG: research design. LB, CS and AS: assay design and software development. LB, CH and VB: experimental work. LB, BP, AS and NG: result analysis and manuscript preparation. NG: research coordination and funding.

## Conflict of Interest Statement

The authors declare that the research was conducted in the absence of any commercial or financial relationships that could be construed as a potential conflict of interest.

## Supplementary Material

FIGURE S1 Ethograms focusing on *abdominal convulsion* for *D. suzukii* **(A)**, *D. biarmipes* **(B)** and *D. melanogaster* **(C)**. All observed transitions between *abdominal convulsion* and sensory sampling-related microbehaviors (green nodes), as well as ovipositor-related microbehaviors (blue nodes) were plotted. Number of transitions between two *abdominal convulsion* events are denoted by black arrows.

FIGURE S2 Relative time and duration of specific microbehaviors. Relative oviposition time (left column) or average duration (right column) for the following microbehaviors: *positioning* **(A,B)**, *egg pushing* **(C,D)**, *protarsal contact* **(E,F)**, *egg expulsion* **(G,H)**, *mesotarsal/metatarsal contact* **(I,J)** and *abdominal convulsion* **(K,L)**. Orange dots on the phylogenetic trees indicate the origin of a divergence (Error bars represent SEM, *n* = 8, stars indicate significant differences between two groups; absence of star: non-significant).

TABLE S1 Start and end time of microbehaviors for 24 individual *Drosophila* females (eight *D. melanogaster*, eight *D. biarmipes* and eight *D. suzukii*).
