# Supplementary figures and images for: Quantitative and Discrete Evolutionary Changes in the Egg-Laying Behavior of Single Drosophila Females
Source: Front Behav Neurosci. 2019 May 29;13:118. doi: 10.3389/fnbeh.2019.00118 (PMC6549446; doi:10.3389/fnbeh.2019.00118)

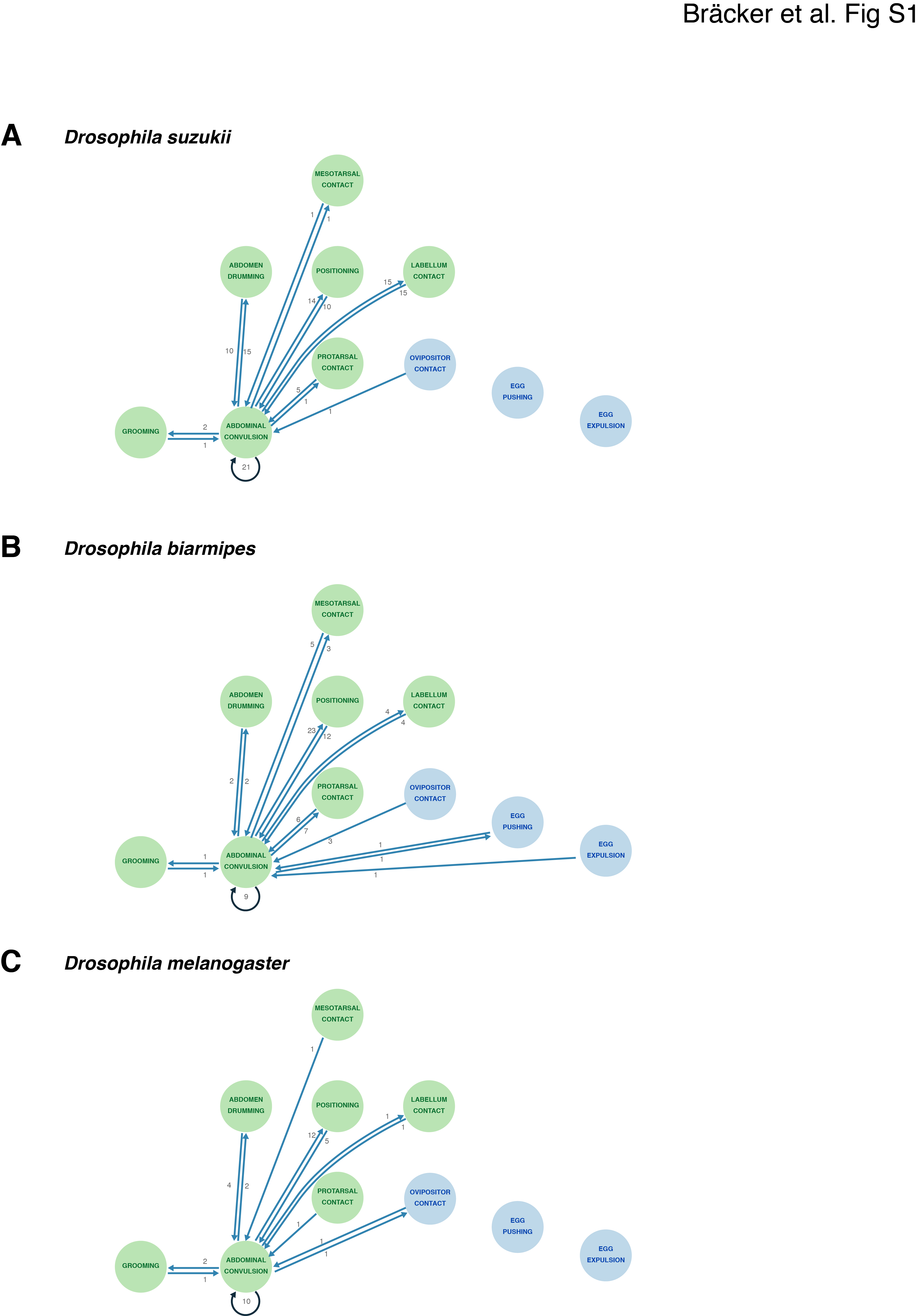

Supplement: FIGURE S1 — Ethograms focusing on abdominal convulsion for D. suzukii (A), D. biarmipes (B) and D. melanogaster (C). All observed transitions between abdominal convulsion and sensory sampling-related microbehaviors (green nodes), as well as ovipositor-related microbehaviors (blue nodes) were plotted. Number of transitions between two abdominal convulsion events are denoted by black arrows. [file Image_1.JPEG]

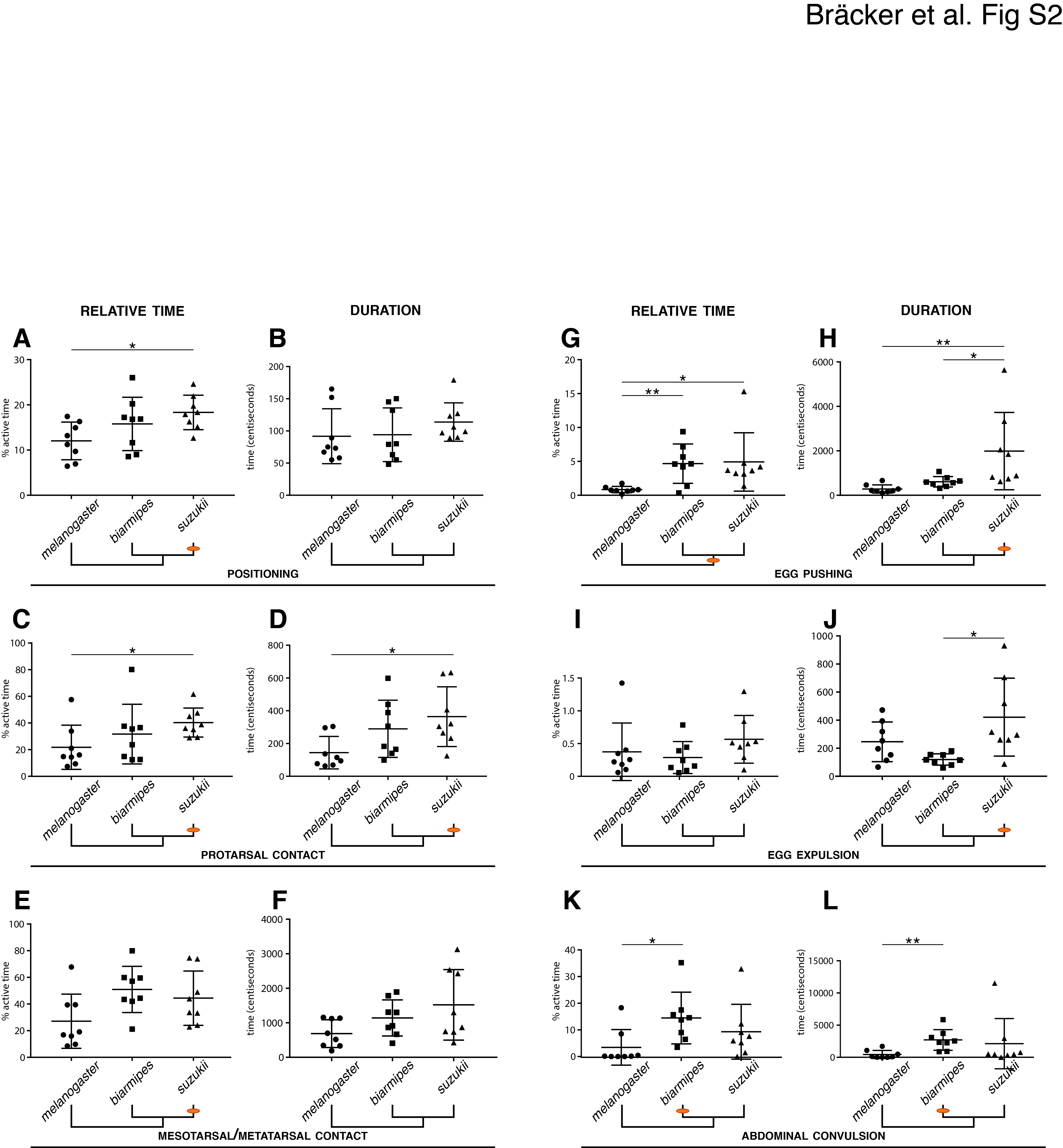

Supplement: FIGURE S2 — Relative time and duration of specific microbehaviors. Relative oviposition time (left column) or average duration (right column) for the following microbehaviors: positioning (A,B), egg pushing (C,D), protarsal contact (E,F), egg expulsion (G,H), mesotarsal/metatarsal contact (I,J) and abdominal convulsion (K,L). Orange dots on the phylogenetic trees indicate the origin of a divergence (Error bars represent SEM, n = 8, stars indicate significant differences between two groups; absence of star: non-significant). [file Image_2.JPEG]
